# 3,5a,9-Trimethyl-8-(2-phenylhydrazin-1-ylidene)-4,5,5a,9b-tetrahydro-3a*H*,8*H*-naphtho[1,2-*b*]furan-2(3*H*)-one

**DOI:** 10.1107/S1600536812023847

**Published:** 2012-06-16

**Authors:** Sammer Yousuf, Syed M. Younas, Nida Ambreen, Khalid M. Khan, Ghulam A. Miana

**Affiliations:** a H.E.J. Research Institute of Chemistry, International Center for Chemical and Biological Sciences, University of Karachi, Karachi 75270, Pakistan; b Department of Chemistry, Allama Iqbal Open University, Islamabad; c Riphah Institute of Pharmaceutical Sciences, Riphah International University, 7th Avenue G-7/4, Islamabad, Pakistan

## Abstract

The title compound, C_21_H_24_N_2_O_2_, is a phenyl hydrazine derivative of the well known anthelminthic agent *α*-santonin, which is composed of three fused rings (benzodieneone, cyclo­hexane and *γ*-lactone). The cyclo­hexa­dienone ring adopts a boat conformation, the cyclo­hexane ring is in a chair conformation and the *trans-*fused γ-lactone ring adopts a C-envelope conformation. In the crystal, mol­ecules are linked by N—H⋯O and C—H⋯O hydrogen bonds, forming chains along the *a* axis.

## Related literature

For the isolation of *α*-santonin, see: Kahler (1830 ▶). For the crystal structure and stereochemistry of *α*-santonin, see: White & Sim (1975 ▶); Coggon & Sim (1969 ▶). For puckering parameters, see: Cremer & Pople (1981 ▶).
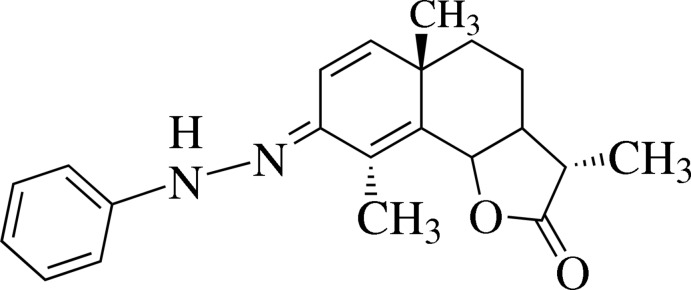


## Experimental

### 

#### Crystal data

C_21_H_24_N_2_O_2_
*M* *_r_* = 336.42
Orthorhombic, 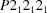

*a* = 10.5104 (12) Å
*b* = 11.5726 (14) Å
*c* = 15.4401 (18) Å
*V* = 1878.0 (4) Å^3^
*Z* = 4
Mo *K*α radiation
μ = 0.08 mm^−1^
*T* = 273 K
0.41 × 0.12 × 0.11 mm

#### Data collection

Bruker SMART APEX CCD area-detector diffractometer
Absorption correction: multi-scan (*SADABS*; Bruker, 2000 ▶) *T*_min_ = 0.969, *T*_max_ = 0.992
11178 measured reflections
1999 independent reflections
1370 reflections with *I* > 2σ(*I*)
*R*_int_ = 0.056

#### Refinement

*R*[*F* ^2^ > 2σ(*F* ^2^)] = 0.043
*wR*(*F* ^2^) = 0.099
*S* = 1.04
1999 reflections
229 parameters
H-atom parameters constrained
Δρ_max_ = 0.12 e Å^−3^
Δρ_min_ = −0.11 e Å^−3^

### 

Data collection: *SMART* (Bruker, 2000 ▶); cell refinement: *SAINT* (Bruker, 2000 ▶); data reduction: *SAINT*; program(s) used to solve structure: *SHELXS97* (Sheldrick, 2008 ▶); program(s) used to refine structure: *SHELXL97* (Sheldrick, 2008 ▶); molecular graphics: *SHELXTL* (Sheldrick, 2008 ▶); software used to prepare material for publication: *SHELXTL*, *PARST* (Nardelli, 1995 ▶) and *PLATON* (Spek, 2009 ▶).

## Supplementary Material

Crystal structure: contains datablock(s) global, I. DOI: 10.1107/S1600536812023847/pv2547sup1.cif

Structure factors: contains datablock(s) I. DOI: 10.1107/S1600536812023847/pv2547Isup2.hkl

Supplementary material file. DOI: 10.1107/S1600536812023847/pv2547Isup3.cml

Additional supplementary materials:  crystallographic information; 3D view; checkCIF report

## Figures and Tables

**Table 1:** Hydrogen-bond geometry (Å, °)

*D*—H⋯*A*	*D*—H	H⋯*A*	*D*⋯*A*	*D*—H⋯*A*
N2—H2*A*⋯O1^i^	0.88	2.12	2.978 (4)	164
C8—H8*A*⋯O1^i^	0.93	2.43	3.290 (4)	153

Symmetry code: (i) 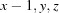.

**Fig. 1.**
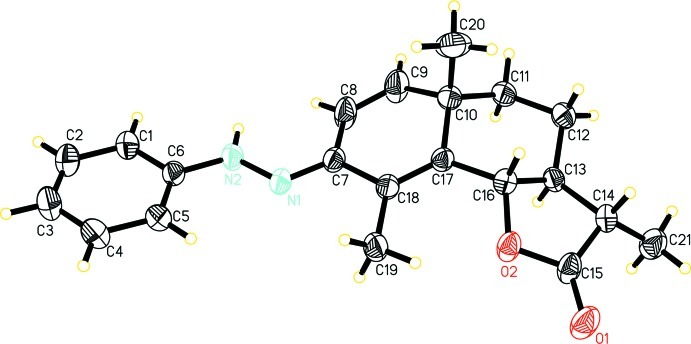
The molecular structure of the title compound with the atom numbering scheme. Displacement ellipsoids are drawn at the 30% probability level. H atoms are presented as small spheres of arbitrary radius.

**Fig. 2.**
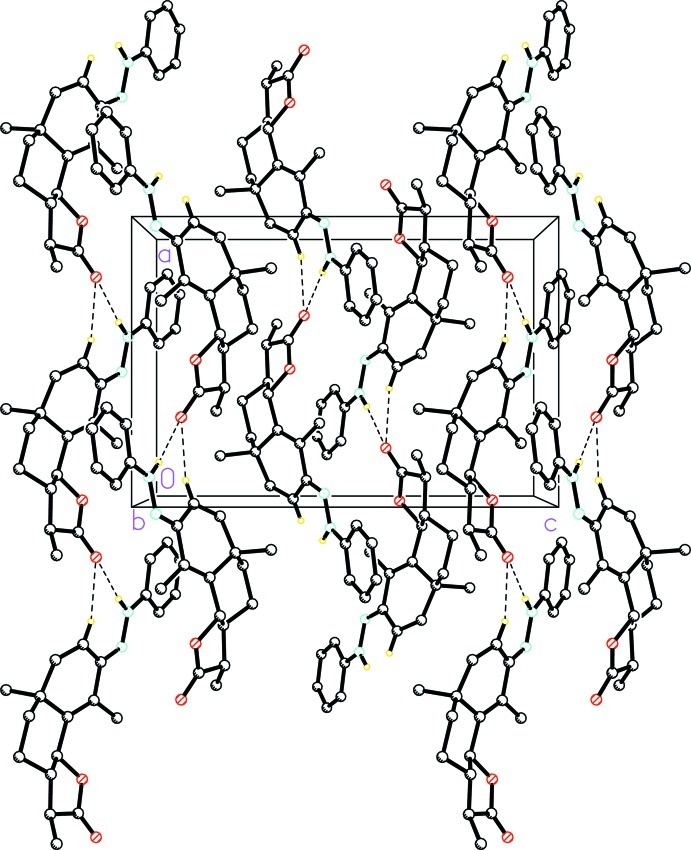
A view of the N—H···O and C—H···O hydrogen bonds (dotted lines) in the crystal structure of the title compound. H atoms non-participating in hydrogen-bonding were omitted for clarity.

## supplementary crystallographic information

### Comment

*α*-Santonin is an anthelminthic agent, first isolated by Kahler in 1830 from *Artemisia santonica*. The title compound is a derivative of *α*-santonin which was synthesized by reacting its dienone with phenyl hydrazine to study its biological activities.

The title molecule (Fig. 1) is composed of a phenyl hydrazine moeity (N1–N2/C1–C6) attached to the *α*-santonin which is composed of three fused rings. The cyclohexadienone ring (C7–C10/C17–C18) adopts a boat conformation C7 and C10 atoms 0.116 (3) and 0.140 (3) Å out of the plane formed by the remaining ring atoms (C8–C9/C17–C18). The cyclohexane ring (C10–C13/C16/C17) adopts a chair conformation with ring puckering parameters (Cremer & Pople, 1981): *Q* = 0.552 (3) Å, θ = 11.3 (3)° and φ = 171.3 (18)°. The *trans* fused γ lactone ring (O2/C13–C16 ) adopts a C13-envelope conformation with C13 0.614 (5) Å out of the plane formed by the rest of the ring atoms. The two methyl substituents on C10 and C14 exist in *axial* and *pseudo equatorial* orientations, respectively. In the crystal structure, the molecules are linked by N2–H2A···O1 and C8–H8A···O1 interactions to form infinite chains running along the *a*-axis (Fig. 2 and Tab. 1). The molecular dimensions in the title compound are similar to those found in structurally related compounds (White & Sim, 1975; Coggon & Sim, 1969). The stereochemistry was assigned on the basis of the published *α*-santonin crystal data (White & Sim, 1975; Coggon & Sim, 1969).

### Experimental

In a 100 ml round bottomed-flask toluene (25 ml) and *α*-santonin (400 mg, 1.6 mmol) were taken and than added phenyl hydrazine (1.1 ml, 11.2 mmol) with contineous stirring. The reaction mixture was refluxed and monitored by TLC. After 24 h the reaction was completed, it was cooled and extracted with water. The organic phase was dried over anhydrous sodium sulfate, filtered and the solvent was evaporated under vacuum on a rotary evaporator. The crude product was chromatographed on a silica gel column using *n*-hexane:ethyl acetate (7:3) as an eluent to obtain pure yellow crystals of the title compound in 80% yield.

### Refinement

All H atoms were positioned geometrically and refined using a riding model, with N—H = 0.88 Å and C—H = 0.95, 0.96, 0.97 and 0.98 Å, for aryl, methyl, methylene and methyne H-atoms, respectively. The *U*_iso_(H) were allowed at 1.5*U*_eq_(C methyl) or 1.2*U*_eq_(C non-methyl). In the absence of sufficient anomalous dispersion effects, an absolute structure was not established in this analysis and 1491 Friedel pairs were merged.

### Crystal data

**Table:**

C_21_H_24_N_2_O_2_	*F*(000) = 720
*M**_r_* = 336.42	*D*_x_ = 1.190 Mg m^−^^3^
Orthorhombic, *P*2_1_2_1_2_1_	Mo *K*α radiation, λ = 0.71073 Å
Hall symbol: P 2ac 2ab	Cell parameters from 1104 reflections
*a* = 10.5104 (12) Å	θ = 2.2–18.1°
*b* = 11.5726 (14) Å	µ = 0.08 mm^−^^1^
*c* = 15.4401 (18) Å	*T* = 273 K
*V* = 1878.0 (4) Å^3^	Block, yellow
*Z* = 4	0.41 × 0.12 × 0.11 mm

### Data collection

**Table:**

Bruker SMART APEX CCD area-detector diffractometer	1999 independent reflections
Radiation source: fine-focus sealed tube	1370 reflections with *I* > 2σ(*I*)
Graphite monochromator	*R*_int_ = 0.056
ω scan	θ_max_ = 25.5°, θ_min_ = 2.2°
Absorption correction: multi-scan (*SADABS*; Bruker, 2000)	*h* = −12→11
*T*_min_ = 0.969, *T*_max_ = 0.992	*k* = −14→14
11178 measured reflections	*l* = −18→18

### Refinement

**Table:**

Refinement on *F*^2^	Secondary atom site location: difference Fourier map
Least-squares matrix: full	Hydrogen site location: inferred from neighbouring sites
*R*[*F*^2^ > 2σ(*F*^2^)] = 0.043	H-atom parameters constrained
*wR*(*F*^2^) = 0.099	*w* = 1/[σ^2^(*F*_o_^2^) + (0.0404*P*)^2^ + 0.0789*P*] where *P* = (*F*_o_^2^ + 2*F*_c_^2^)/3
*S* = 1.04	(Δ/σ)_max_ < 0.001
1999 reflections	Δρ_max_ = 0.12 e Å^−^^3^
229 parameters	Δρ_min_ = −0.11 e Å^−^^3^
0 restraints	Absolute structure: Flack (1983), 1491 Friedel pairs
Primary atom site location: structure-invariant direct methods	Flack parameter: 0 (10)

### Special details

**Table:**

Geometry. All e.s.d.'s (except the e.s.d. in the dihedral angle between two l.s. planes) are estimated using the full covariance matrix. The cell e.s.d.'s are taken into account individually in the estimation of e.s.d.'s in distances, angles and torsion angles; correlations between e.s.d.'s in cell parameters are only used when they are defined by crystal symmetry. An approximate (isotropic) treatment of cell e.s.d.'s is used for estimating e.s.d.'s involving l.s. planes.
Refinement. Refinement of *F*^2^ against ALL reflections. The weighted *R*-factor *wR* and goodness of fit *S* are based on *F*^2^, conventional *R*-factors *R* are based on *F*, with *F* set to zero for negative *F*^2^. The threshold expression of *F*^2^ > σ(*F*^2^) is used only for calculating *R*-factors(gt) *etc*. and is not relevant to the choice of reflections for refinement. *R*-factors based on *F*^2^ are statistically about twice as large as those based on *F*, and *R*- factors based on ALL data will be even larger.

### Fractional atomic coordinates and isotropic or equivalent isotropic displacement parameters (Å^2^)

**Table:**

	*x*	*y*	*z*	*U*_iso_*/*U*_eq_	
O1	0.6908 (2)	0.2836 (2)	0.39485 (19)	0.0860 (9)	
O2	0.4891 (2)	0.31375 (18)	0.35869 (14)	0.0599 (6)	
N1	0.0266 (2)	0.4301 (2)	0.44717 (16)	0.0536 (7)	
N2	−0.1011 (3)	0.4328 (2)	0.46280 (17)	0.0604 (8)	
H2A	−0.1513	0.3781	0.4430	0.073*	
C1	−0.2860 (3)	0.5333 (3)	0.5113 (2)	0.0641 (10)	
H1B	−0.3359	0.4719	0.4925	0.077*	
C2	−0.3417 (4)	0.6278 (4)	0.5491 (2)	0.0807 (12)	
H2B	−0.4294	0.6290	0.5569	0.097*	
C3	−0.2708 (5)	0.7203 (4)	0.5756 (3)	0.0867 (13)	
H3A	−0.3099	0.7843	0.6006	0.104*	
C4	−0.1405 (4)	0.7178 (3)	0.5648 (2)	0.0763 (11)	
H4A	−0.0915	0.7804	0.5825	0.092*	
C5	−0.0824 (4)	0.6230 (3)	0.52795 (19)	0.0615 (10)	
H5A	0.0055	0.6219	0.5212	0.074*	
C6	−0.1541 (3)	0.5297 (3)	0.50100 (19)	0.0525 (8)	
C7	0.0722 (3)	0.3454 (3)	0.4016 (2)	0.0514 (8)	
C8	−0.0011 (3)	0.2516 (3)	0.3661 (3)	0.0810 (12)	
H8A	−0.0833	0.2388	0.3862	0.097*	
C9	0.0463 (3)	0.1832 (4)	0.3053 (3)	0.0892 (14)	
H9A	−0.0044	0.1232	0.2849	0.107*	
C10	0.1765 (3)	0.1967 (3)	0.2675 (2)	0.0590 (9)	
C11	0.2292 (3)	0.0739 (3)	0.2527 (2)	0.0661 (10)	
H11A	0.2156	0.0288	0.3049	0.079*	
H11B	0.1812	0.0377	0.2064	0.079*	
C12	0.3705 (3)	0.0699 (3)	0.2297 (2)	0.0677 (10)	
H12A	0.3996	−0.0096	0.2271	0.081*	
H12B	0.3847	0.1055	0.1736	0.081*	
C13	0.4414 (3)	0.1346 (3)	0.29870 (19)	0.0468 (8)	
H13A	0.4208	0.0986	0.3544	0.056*	
C14	0.5848 (3)	0.1514 (3)	0.2955 (2)	0.0528 (9)	
H14A	0.6076	0.1756	0.2367	0.063*	
C15	0.5995 (3)	0.2533 (3)	0.3539 (2)	0.0585 (9)	
C16	0.3967 (3)	0.2589 (3)	0.30242 (19)	0.0485 (8)	
H16A	0.4075	0.2919	0.2444	0.058*	
C17	0.2590 (3)	0.2721 (3)	0.32667 (19)	0.0462 (8)	
C18	0.2099 (3)	0.3460 (2)	0.3847 (2)	0.0489 (8)	
C19	0.2862 (3)	0.4331 (3)	0.4352 (2)	0.0676 (10)	
H19A	0.3618	0.4525	0.4036	0.101*	
H19B	0.3092	0.4008	0.4903	0.101*	
H19C	0.2361	0.5015	0.4440	0.101*	
C20	0.1620 (4)	0.2591 (4)	0.1789 (2)	0.0962 (14)	
H20A	0.1238	0.3335	0.1876	0.144*	
H20B	0.1089	0.2137	0.1414	0.144*	
H20C	0.2443	0.2685	0.1528	0.144*	
C21	0.6686 (3)	0.0503 (3)	0.3200 (3)	0.0778 (11)	
H21A	0.7553	0.0758	0.3243	0.117*	
H21B	0.6622	−0.0086	0.2764	0.117*	
H21C	0.6416	0.0196	0.3747	0.117*	

### Atomic displacement parameters (Å^2^)

**Table:**

	*U*^11^	*U*^22^	*U*^33^	*U*^12^	*U*^13^	*U*^23^
O1	0.0446 (16)	0.0785 (18)	0.135 (2)	−0.0044 (14)	−0.0081 (16)	−0.0317 (17)
O2	0.0391 (13)	0.0468 (12)	0.0939 (16)	−0.0028 (11)	0.0040 (13)	−0.0164 (12)
N1	0.0413 (17)	0.0588 (16)	0.0606 (16)	0.0050 (14)	0.0022 (14)	−0.0076 (15)
N2	0.0418 (17)	0.0653 (18)	0.0742 (19)	0.0057 (15)	0.0050 (15)	−0.0194 (16)
C1	0.054 (2)	0.071 (2)	0.067 (2)	0.0120 (19)	0.0064 (19)	−0.007 (2)
C2	0.065 (3)	0.094 (3)	0.083 (3)	0.022 (2)	0.011 (2)	−0.013 (3)
C3	0.094 (4)	0.082 (3)	0.084 (3)	0.030 (3)	0.007 (3)	−0.022 (2)
C4	0.090 (3)	0.073 (3)	0.065 (2)	0.009 (2)	−0.004 (2)	−0.018 (2)
C5	0.066 (3)	0.068 (2)	0.051 (2)	0.008 (2)	−0.0021 (18)	−0.0071 (18)
C6	0.053 (2)	0.061 (2)	0.0441 (18)	0.0110 (18)	0.0035 (17)	−0.0011 (17)
C7	0.040 (2)	0.052 (2)	0.062 (2)	0.0034 (15)	0.0010 (16)	−0.0070 (18)
C8	0.037 (2)	0.078 (3)	0.128 (3)	−0.003 (2)	0.009 (2)	−0.039 (3)
C9	0.042 (2)	0.088 (3)	0.138 (4)	−0.009 (2)	0.000 (2)	−0.055 (3)
C10	0.050 (2)	0.061 (2)	0.066 (2)	0.0008 (18)	−0.0068 (18)	−0.0180 (18)
C11	0.056 (2)	0.067 (2)	0.075 (2)	−0.0019 (19)	−0.0070 (19)	−0.0314 (19)
C12	0.064 (3)	0.067 (2)	0.073 (2)	0.0121 (19)	−0.005 (2)	−0.027 (2)
C13	0.046 (2)	0.0500 (18)	0.0440 (17)	0.0033 (14)	0.0070 (16)	−0.0049 (16)
C14	0.044 (2)	0.057 (2)	0.0566 (19)	0.0050 (16)	0.0108 (17)	−0.0030 (17)
C15	0.038 (2)	0.054 (2)	0.084 (2)	−0.0033 (18)	0.0100 (19)	−0.006 (2)
C16	0.0470 (19)	0.0466 (18)	0.0518 (17)	0.0021 (15)	0.0030 (16)	0.0021 (17)
C17	0.0387 (19)	0.0442 (18)	0.0557 (18)	−0.0011 (14)	−0.0006 (15)	−0.0051 (16)
C18	0.043 (2)	0.0490 (19)	0.0546 (19)	0.0014 (16)	−0.0012 (16)	−0.0066 (16)
C19	0.049 (2)	0.069 (2)	0.085 (2)	0.0004 (19)	0.004 (2)	−0.030 (2)
C20	0.103 (3)	0.100 (3)	0.085 (3)	0.018 (3)	−0.043 (3)	−0.016 (3)
C21	0.065 (2)	0.063 (2)	0.105 (3)	0.014 (2)	−0.007 (2)	−0.020 (2)

### Geometric parameters (Å, º)

**Table:**

O1—C15	1.202 (4)	C10—C20	1.554 (5)
O2—C15	1.357 (4)	C11—C12	1.528 (4)
O2—C16	1.449 (4)	C11—H11A	0.9700
N1—C7	1.298 (4)	C11—H11B	0.9700
N1—N2	1.365 (3)	C12—C13	1.501 (4)
N2—C6	1.385 (4)	C12—H12A	0.9700
N2—H2A	0.8779	C12—H12B	0.9700
C1—C2	1.371 (4)	C13—C16	1.515 (4)
C1—C6	1.396 (4)	C13—C14	1.520 (4)
C1—H1B	0.9300	C13—H13A	0.9800
C2—C3	1.367 (6)	C14—C15	1.492 (4)
C2—H2B	0.9300	C14—C21	1.513 (4)
C3—C4	1.380 (5)	C14—H14A	0.9800
C3—H3A	0.9300	C16—C17	1.502 (4)
C4—C5	1.378 (5)	C16—H16A	0.9800
C4—H4A	0.9300	C17—C18	1.341 (4)
C5—C6	1.381 (5)	C18—C19	1.506 (4)
C5—H5A	0.9300	C19—H19A	0.9600
C7—C8	1.440 (4)	C19—H19B	0.9600
C7—C18	1.471 (4)	C19—H19C	0.9600
C8—C9	1.325 (5)	C20—H20A	0.9600
C8—H8A	0.9300	C20—H20B	0.9600
C9—C10	1.495 (5)	C20—H20C	0.9600
C9—H9A	0.9300	C21—H21A	0.9600
C10—C17	1.533 (4)	C21—H21B	0.9600
C10—C11	1.542 (5)	C21—H21C	0.9600
			
C15—O2—C16	108.3 (2)	C11—C12—H12B	110.2
C7—N1—N2	118.4 (3)	H12A—C12—H12B	108.5
N1—N2—C6	119.3 (3)	C12—C13—C16	110.3 (3)
N1—N2—H2A	120.9	C12—C13—C14	122.2 (3)
C6—N2—H2A	119.4	C16—C13—C14	100.8 (2)
C2—C1—C6	119.8 (4)	C12—C13—H13A	107.6
C2—C1—H1B	120.1	C16—C13—H13A	107.6
C6—C1—H1B	120.1	C14—C13—H13A	107.6
C3—C2—C1	121.3 (4)	C15—C14—C21	113.5 (3)
C3—C2—H2B	119.4	C15—C14—C13	100.6 (3)
C1—C2—H2B	119.4	C21—C14—C13	118.1 (3)
C2—C3—C4	119.2 (4)	C15—C14—H14A	108.0
C2—C3—H3A	120.4	C21—C14—H14A	108.0
C4—C3—H3A	120.4	C13—C14—H14A	108.0
C5—C4—C3	120.5 (4)	O1—C15—O2	120.2 (3)
C5—C4—H4A	119.8	O1—C15—C14	129.2 (3)
C3—C4—H4A	119.8	O2—C15—C14	110.6 (3)
C4—C5—C6	120.3 (4)	O2—C16—C17	116.9 (2)
C4—C5—H5A	119.8	O2—C16—C13	103.3 (2)
C6—C5—H5A	119.8	C17—C16—C13	113.9 (3)
C5—C6—N2	122.9 (3)	O2—C16—H16A	107.4
C5—C6—C1	119.0 (3)	C17—C16—H16A	107.4
N2—C6—C1	118.1 (3)	C13—C16—H16A	107.4
N1—C7—C8	125.3 (3)	C18—C17—C16	127.0 (3)
N1—C7—C18	117.1 (3)	C18—C17—C10	122.9 (3)
C8—C7—C18	117.6 (3)	C16—C17—C10	109.8 (3)
C9—C8—C7	121.2 (3)	C17—C18—C7	119.6 (3)
C9—C8—H8A	119.4	C17—C18—C19	124.6 (3)
C7—C8—H8A	119.4	C7—C18—C19	115.8 (3)
C8—C9—C10	123.9 (4)	C18—C19—H19A	109.5
C8—C9—H9A	118.0	C18—C19—H19B	109.5
C10—C9—H9A	118.0	H19A—C19—H19B	109.5
C9—C10—C17	110.2 (3)	C18—C19—H19C	109.5
C9—C10—C11	106.8 (3)	H19A—C19—H19C	109.5
C17—C10—C11	114.2 (3)	H19B—C19—H19C	109.5
C9—C10—C20	107.6 (3)	C10—C20—H20A	109.5
C17—C10—C20	108.4 (3)	C10—C20—H20B	109.5
C11—C10—C20	109.4 (3)	H20A—C20—H20B	109.5
C12—C11—C10	114.3 (3)	C10—C20—H20C	109.5
C12—C11—H11A	108.7	H20A—C20—H20C	109.5
C10—C11—H11A	108.7	H20B—C20—H20C	109.5
C12—C11—H11B	108.7	C14—C21—H21A	109.5
C10—C11—H11B	108.7	C14—C21—H21B	109.5
H11A—C11—H11B	107.6	H21A—C21—H21B	109.5
C13—C12—C11	107.6 (3)	C14—C21—H21C	109.5
C13—C12—H12A	110.2	H21A—C21—H21C	109.5
C11—C12—H12A	110.2	H21B—C21—H21C	109.5
C13—C12—H12B	110.2		
			
C7—N1—N2—C6	−171.3 (3)	C16—O2—C15—C14	−1.8 (3)
C6—C1—C2—C3	1.4 (6)	C21—C14—C15—O1	28.7 (5)
C1—C2—C3—C4	−0.7 (6)	C13—C14—C15—O1	155.9 (4)
C2—C3—C4—C5	−0.1 (6)	C21—C14—C15—O2	−150.0 (3)
C3—C4—C5—C6	0.3 (5)	C13—C14—C15—O2	−22.8 (3)
C4—C5—C6—N2	179.3 (3)	C15—O2—C16—C17	151.9 (3)
C4—C5—C6—C1	0.3 (5)	C15—O2—C16—C13	26.1 (3)
N1—N2—C6—C5	−3.0 (5)	C12—C13—C16—O2	−169.4 (2)
N1—N2—C6—C1	176.0 (3)	C14—C13—C16—O2	−38.9 (3)
C2—C1—C6—C5	−1.2 (5)	C12—C13—C16—C17	62.9 (3)
C2—C1—C6—N2	179.8 (3)	C14—C13—C16—C17	−166.7 (3)
N2—N1—C7—C8	−0.7 (5)	O2—C16—C17—C18	13.5 (5)
N2—N1—C7—C18	179.0 (3)	C13—C16—C17—C18	133.9 (3)
N1—C7—C8—C9	165.1 (4)	O2—C16—C17—C10	−172.9 (2)
C18—C7—C8—C9	−14.7 (5)	C13—C16—C17—C10	−52.4 (3)
C7—C8—C9—C10	−0.8 (7)	C9—C10—C17—C18	−21.6 (5)
C8—C9—C10—C17	17.8 (6)	C11—C10—C17—C18	−141.8 (3)
C8—C9—C10—C11	142.3 (4)	C20—C10—C17—C18	95.9 (4)
C8—C9—C10—C20	−100.2 (5)	C9—C10—C17—C16	164.4 (3)
C9—C10—C11—C12	−169.5 (3)	C11—C10—C17—C16	44.3 (4)
C17—C10—C11—C12	−47.5 (4)	C20—C10—C17—C16	−78.0 (3)
C20—C10—C11—C12	74.3 (4)	C16—C17—C18—C7	−178.8 (3)
C10—C11—C12—C13	54.0 (4)	C10—C17—C18—C7	8.3 (5)
C11—C12—C13—C16	−60.2 (4)	C16—C17—C18—C19	1.7 (5)
C11—C12—C13—C14	−178.2 (3)	C10—C17—C18—C19	−171.2 (3)
C12—C13—C14—C15	159.0 (3)	N1—C7—C18—C17	−169.1 (3)
C16—C13—C14—C15	36.5 (3)	C8—C7—C18—C17	10.7 (5)
C12—C13—C14—C21	−76.9 (4)	N1—C7—C18—C19	10.4 (4)
C16—C13—C14—C21	160.6 (3)	C8—C7—C18—C19	−169.8 (3)
C16—O2—C15—O1	179.4 (3)		

### Hydrogen-bond geometry (Å, º)

**Table:**

*D*—H···*A*	*D*—H	H···*A*	*D*···*A*	*D*—H···*A*
N2—H2*A*···O1^i^	0.88	2.12	2.978 (4)	164
C8—H8*A*···O1^i^	0.93	2.43	3.290 (4)	153

Symmetry code: (i) *x*−1, *y*, *z*.
